# Left Atrial Dilatation and Reduced Left Ventricular Ejection Fraction Are Associated With Cardioembolic Stroke

**DOI:** 10.3389/fneur.2021.680651

**Published:** 2021-09-13

**Authors:** Maryam Hosseini Farahabadi, Shadi Milani-Nejad, Shimeng Liu, Wengui Yu, Mohammad Shafie

**Affiliations:** ^1^Department of Neurology, University of California, Irvine, Irvine, CA, United States; ^2^Department of Neurology, Beijing Tiatan Hospital, Capital Medical University, Beijing, China

**Keywords:** cardioembolic stroke, left atrial dilatation, reduced ejection fraction, atrial fibrillation, heart failure

## Abstract

**Objective:** Left atrial (LA) dilatation and heart failure are independent risk factors for ischemic stroke. The goal of this study is to evaluate the association between LA dilatation and reduced left ventricular ejection fraction (EF) with cardioembolic stroke.

**Methods:** Four hundred fifty-three patients with ischemic stroke admitted to the University of California, Irvine between 2016 and 2017 were included based on the following criteria: age >18 and availability of echocardiogram. Stroke was categorized into cardioembolic and non-cardioembolic. EF was categorized into normal: 52–72% (male), 54–74% (female), mildly abnormal: 41–51% (male), 41–53% (female), moderately abnormal: 30–40%, and severely abnormal: <30%. LA volume was categorized into normal (≤34 ml/m^2^) vs. enlarged (≥35 ml/m^2^). Other variables included gender, hypertension [systolic blood pressure (SBP) ≥ 140 or diastolic blood pressure (DBP) ≥ 90], and known history of atrial fibrillation (Afib).

**Results:** Two hundred eighteen patients had cardioembolic, and 235 had non-cardioembolic stroke. Among patients with cardioembolic stroke, 49 (22.4%) and 142 (65%) had reduced EF and enlarged LA, respectively, as compared with 19 (8.1%) and 65 (27.7%) patients with non-cardioembolic stroke (*p* < 0.0001). The odds of cardioembolic stroke were 2.0 (95% CI: 0.1–6.0) and 8.8 times (95% CI: 1.9–42.3) higher in patients with moderately and severely reduced EF, respectively, than in patients with normal EF. The odds of cardioembolic stroke was 2.4 times (95% CI: 1.5–3.9) higher in patients with enlarged LA than in patients with normal LA size. Compared with patients with normal LA and EF, patients with combined enlarged LA and reduced EF had significantly higher rates of Afib (43.4 vs. 9.0%, *p* < 0.0001) and cardioembolic stroke (78.3 vs. 43.4%, *p* < 0.0001).

**Conclusions:** LA dilatation along with reduced EF is a reliable predictor of Afib and cardioembolic stroke. Further studies are warranted to determine the benefit of anticoagulation for secondary stroke prevention in such patient population.

## Introduction

The etiology of ischemic stroke impacts prognosis and management. Based on the TOAST criteria, ischemic stroke is classified into five categories: cardioembolic, large-artery atherosclerosis, small vessel occlusion, stroke of other determined etiology, and stroke of undetermined etiology. Cardioembolic stroke includes patients with arterial occlusion due to an embolus presumably arising in the heart. Up to 25% of ischemic strokes are cardioembolic in nature with atrial fibrillation (Afib) being the most common underlying etiology (1–4). Detection and diagnosis of Afib often requires long-term monitoring, which is costly with variable detection rates reaching only 30% in 3 years (5, 6). In addition to abnormal atrial rhythm detection, structural assessment of atrial size may prove as a potential diagnostic tool. In particular, left atrial (LA) volume index > 32 ml/m^2^ in patients without Afib has been demonstrated to be predictive of first-ever ischemic stroke (7). Additionally, LA enlargement has been shown as an echocardiographic indicator of Afib (8, 9). Studies have shown that in the presence of LA dilatation, the likelihood of Afib detection will be higher, and that every 5 mm incremental increase in LA size raises the risk of developing Afib by 39% (10–12). Furthermore, studies have shown that LA dilatation is correlated with cardioembolic compared with atherosclerotic stroke (13).

Heart failure (HF) is another potential independent cardioembolic risk factor accounting for etiology of ~9% of ischemic strokes with a 9–10% risk of recurrent stroke per year in patients with HF (14, 15). Moreover, these patients are at a greater risk of developing Afib, and conversely, patients with Afib are more likely to develop HF (16). Even among patients with cryptogenic stroke, which makes up to 20–30% of all ischemic strokes, low burden occult Afib has been shown to be one of the underlying etiologies with its frequency increasing in patients older than 60 years of age (17). Discovery of the culprit cardioembolic source despite the most thorough diagnostic testing often remains a challenge; however, its identification is imperative as it will ultimately guide the decisions regarding anticoagulation vs. antiplatelet treatment for secondary stroke prevention.

Of note, patients with HF often have other comorbidities, such as hypertension or ischemic heart disease, which also increase the risk of ischemic stroke (14, 18). However, even after adjusting for those risk factors, there remains 2–3 times higher risk of stroke in patients with HF (19). Additionally, ischemic stroke in HF patients is associated with higher mortality rate and longer hospital length of stay (15). Investigations in HF patients with reduced ejection fraction (EF) without Afib have identified moderate and severe HF [New York Heart Association (NYHA) classes III and IV], insulin-dependent diabetes mellitus, high body mass index, and previous history of stroke as independent stroke risk factors (20). Stratification based on these risk factors demonstrated that these patients may have a rate of stroke risk approximating patients with Afib and not treated with anticoagulation. Accordingly, in patients with a combination of HF and Afib, even paroxysmal in nature, the risk of stroke is higher, and therefore consideration of these risk factors will allow for individualization of stroke prevention measures.

The Heart Failure Society of America recommends consideration of anticoagulation for stroke prevention in patients with EF below 35%. However, studies have failed to prove the benefit of anticoagulation over antiplatelet therapy in this patient population (21). It is unclear if patients with LA enlargement and low EF without Afib will benefit from anticoagulation therapy. In this study, we aimed to evaluate the association between LA dilatation and reduced EF with cardioembolic stroke.

## Materials and Methods

### Ethics

Ethics approval for the study including human participants was obtained from the UCI institutional review board.

**Figure 1:**
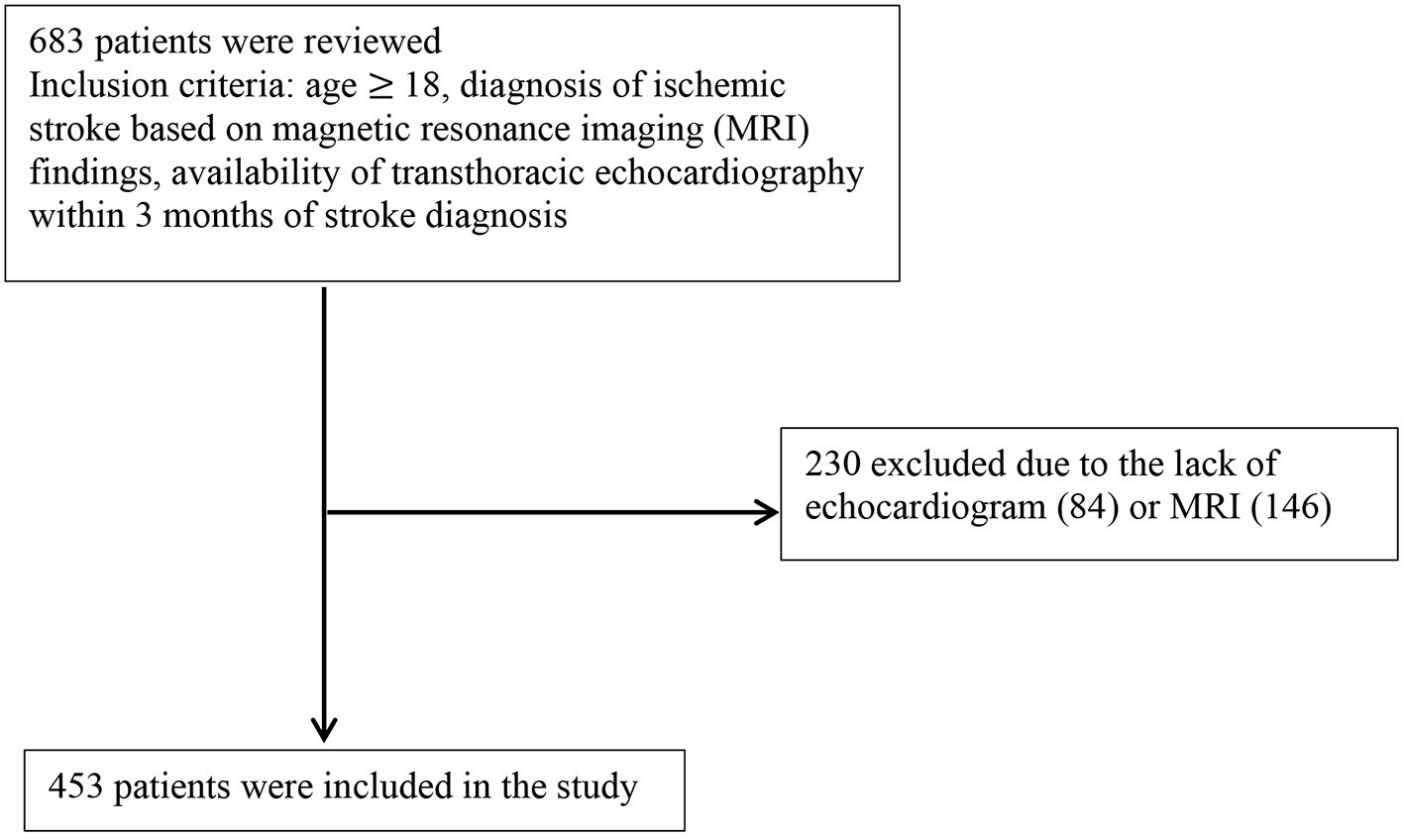
Flow diagram for inclusion and exclusion criteria.

Six hundred eighty-three consecutive patients diagnosed with ischemic stroke between 2016 and 2017 at the University of California, Irvine were reviewed. Inclusion criteria included: age ≥ 18, diagnosis of ischemic stroke based on magnetic resonance imaging (MRI) findings, and availability of transthoracic echocardiography (TTE) within 3 months of stroke diagnosis. As shown in Figure 1, from 683 records, 230 were excluded due to lack of MRI or echocardiogram.

The diagnostic work-up for all ischemic stroke patients typically involved MRI of the brain if no contraindications, vessel imaging with either computed tomography angiography (CTA) or magnetic resonance angiography (MRA), TTE, transesophageal echocardiography (TEE) in cases indicated after TTE, and continuous cardiac telemetry monitoring for a minimum of 24–48 h. Further work-up for underlying hypercoagulable state, hematological, rheumatological, or other etiologies was pursued per stroke team discretion.

Stroke was dichotomized into cardioembolic (based on MRI findings and identified cardiac source including: evidence of intracardiac thrombi or endocarditis, history of Afib, or if there was high suspicion for cardioembolic stroke) vs. non-cardioembolic (atheroembolic, small-vessel, stroke of other determined etiology, or non-embolic stroke of undetermined source based on the MRI findings and other stroke work-up). LA volume was measured with the biplane area-length method and categorized as 34 ml/m^2^ or lower (normal) vs. 35 or greater ml/m^2^ (abnormal). Other variables that were taken into consideration included: gender, blood pressure that was dichotomized into hypertension defined as systolic blood pressure (SBP) ≥ 140 mmHg or diastolic blood pressure (DBP) ≥ 90 mmHg vs. normal blood pressure defined as SBP < 140 mmHg and DBP < 90 mmHg upon presentation, EF: 52–72% normal (male), 54–74% (female), 41–51% mildly abnormal (male), 41–53% (female), 30–40% moderately abnormal (same in both genders), <30 severely abnormal (same in both genders), and known history of Afib.

Continuous variables including age and National Institute of Health Stroke Scale (NIHSS) score were presented as median + IQR using the Kruskal–Wallis tests as they were not normally distributed. Chi-square test was used to investigate the distribution of the categorical variables among patients with cardioembolic vs. non-cardioembolic stroke, patients with Afib vs. those without Afib, and also those with reduced EF and LA dilatation vs. normal EF and LA size. Logistic regression model was employed to determine the association between variables and stroke types, while non-cardioembolic stroke was considered as the control group. All statistical analyses were performed using SAS version 9.4. Alpha is 0.05, or a *p*-value < 0.05 is considered statistically significant.

## Results

Demographic characteristics of participants are presented in Table 1. From a total of 453 patients, 218 were categorized into cardioembolic and 235 into non-cardioembolic strokes. Patients with cardioembolic stroke were significantly older with a median age of 72.9 years (vs. 65.2 years, *p* < 0.0001). There was no difference in terms of gender distribution or prevalence of hypertension between the two categories of cardioembolic and non-cardioembolic strokes.

**Table 1:** Demographic characteristics based on cardioembolic vs. non-cardioembolic stroke.

	**All patients (*N* = 453)**	**Non-cardioembolic (*N* = 235, 51.9%)**	**Cardioembolic (*N* = 218, 48.1%)**	***p*-value**
Age, median (IQR)	68.9 (23)	65.2 (23)	72.9 (20)	<0.0001
Gender				
Male	255 (56.3%)	139 (59.1%)	116 (53.2%)	0.20
HTN				
SBP ≥ 140 or DBP ≥ 90	365 (80.5%)	198 (84.3%)	170 (78.0%)	0.08
Atrial fibrillation	112 (24.8%)	9 (3.8%)	103 (47.2%)	<0.0001
EF category, median (IQR)	33 (19)			<0.0001
Normal		216 (91.9%)	169 (77.5%)	
Mildly decreased		10 (4.3%)	16 (7.3%)	
Moderately decreased		7 (3.0%)	16 (7.3%)	
Severely decreased		2 (0.8%)	17 (7.8%)	
Enlarged left atrium	207 (45.7%)	65 (27.7%)	142 (65.1%)	<0.0001
NIHSS, median (IQR)	6 (12)	4 (5)	11 (15)	<0.0001

*EF, ejection fraction; HTN, hypertension; IQR, interquartile range; SBP, systolic blood pressure; DBP, diastolic blood pressure*.

While 103 (47.2%) patients with cardioembolic stroke had known history of Afib, this was only 9 (3.8%) among patients with non-cardioembolic stroke (*p* < 0.0001). Among patients with non-cardioembolic stroke, 91.9% had normal EF compared with 77.5% of patients with cardioembolic stroke. Among patients with non-cardioembolic stroke, 4.3, 3.0, and 0.8% had mildly, moderately, and severely decreased EFs, respectively, vs. corresponding 7.3, 7.3, and 7.8%, respectively, in patients with cardioembolic stroke (*p* < 0.001). The prevalence of enlarged LA was 27.7% in non-cardioembolic stroke vs. 65.1% in cardioembolic stroke (*p* < 0.0001). The median NIHSS scores in cardioembolic and non-cardioembolic stroke were 12 and 6, respectively (*p* < 0.0001).

Table 2 demonstrates the association between cardioembolic stroke and other covariates including age, gender, blood pressure, Afib, EF and enlarged left atrium. Cardioembolic stroke is weakly associated with older age (95% CI: 1.002–1.035). Patients with severely reduced left ventricular function (EF < 30%) are at 8.85 times risk of developing cardioembolic stroke compared with those with normal EF (95% CI: 1.852–42.266). Patients with enlarged left atrium have 2.44 odds of cardioembolic stroke compared with those with normal LA size (95% CI: 1.519–3.929). History of Afib is associated with significantly higher odds of cardioembolic stroke (95% CI: 7.005–31.204). Gender and history of hypertension were not associated with cardioembolic stroke after adjusting for other variables.

**Table 2:** Multiple logistic regression model.

**Covariate**	**Odds ratio**	**95% confidence interval**
Age	1.02	1.002–1.035
Gender		
Male	1.08	0.676–1.732
HTN		
SBP ≥ 140 or DBP ≥ 90	0.73	0.408–1.319
Atrial fibrillation	14.78	7.005–31.204
EF category		
Mildly decreased	1.30	0.469–3.584
Moderately decreased	2.04	0.070–5.959
Severely decreased	8.85	1.852–42.266
Enlarged LA	2.44	1.519–3.929

*The association between cardioembolic stroke and other covariates. EF, ejection fraction; HTN, hypertension; LA, left atrium; SBP, systolic blood pressure; DBP, diastolic blood pressure*.

Table 3 shows the demographic characteristics of patients with known history of Afib and those without Afib. Patients with Afib are significantly older (78 vs. 67 years; *p* < 0.001), less likely to be male (47.3 vs. 59.2%; *p* = 0.03), less likely to have normal EF (75.9 vs. 88.0%; *p* = 0.01), and more likely to have enlarged left atrium (75.9 vs. 35.8%; *p* < 0.0001) than patients without Afib.

**Table 3:** Demographic characteristics of patients with Afib vs. those without Afib.

	***N* = 453**	**Non-AF (*N* = 341)**	**AF (*N* = 112)**	***p*-value**
Age, median (IQR)	72 (23)	67 (22)	78 (16)	<0.0001
Gender				
Male	255	202 (59.2%)	53 (47.3%)	0.03
HTN				
SBP ≥ 140 or DBP ≥ 90	368	282 (82.7%)	86 (76.8%)	0.16
EF category	453			0.01
Normal		300 (88.0%)	85 (75.9%)	
Mildly decreased		14 (4.11%)	12 (10.71%)	
Moderately decreased		14 (4.11%)	9 (8.04%)	
Severely decreased		13 (3.81%)	6 (5.36%)	
Enlarged LA	207	122 (35.8%)	85 (75.9%)	<0.0001

*Afib, atrial fibrillation; EF, ejection fraction; HTN, hypertension; SBP, systolic blood pressure; DBP, diastolic blood pressure*.

To investigate the association of combined LA dilatation and reduced EF with Afib and cardioembolic stroke, we divided patients into two groups: normal LA size and normal EF vs. enlarged LA and reduced EF. In this subgroup analysis as shown in Table 4, patients with combined enlarged LA and reduced EF had significantly higher rates of Afib (43.4 vs. 8.9%, *p* < 0.0001) and cardioembolic stroke (78.3 vs. 28.1%, *p* < 0.0001) than patients with normal LA and EF.

**Table 4:** LA enlargement and reduced EF in patients with Afib and cardioembolic stroke.

	***N* = 270**	**Normal LA and EF (*N* = 224)**	**Enlarged LA and reduced EF (*N* = 46)**	***p*-value**
Afib	40	20 (8.9%)	20 (43.4%)	<0.0001
Cardioembolic stroke	99	63 (28.1%)	36 (78.3%)	<0.0001

*Afib, atrial fibrillation; EF, ejection fraction; LA, left atrium*.

## Discussion

In this retrospective cohort study, we investigated the association of enlarged LA and reduced left ventricular EF with cardioembolic stroke. This analysis revealed that patients with enlarged LA and severely reduced EF (<30%) are independently associated with 2.4 and 8.8 times higher, respectively, risks of developing cardioembolic stroke than patients with normal values of these parameters. Also, a greater percentage of patients with enlarged LA and reduced EF had Afib and cardioembolic stroke when compared with those with normal LA size and normal EF.

In this study, we used the biplane area-length method to calculate the LA volume, which has been shown to be a more accurate measurement than LA diameter with excellent inter-rater reliability (interclass correlation 0.94) (22). LA enlargement in patients without prior history of Afib is an independent risk factor for first time and recurrent stroke (8, 9). Additionally, LA diameter > 0.5 mm has been shown to be correlated with a 4-fold increased risk of new-onset Afib (12, 23).

Patients with Afib are more likely to develop HF, and conversely, HF patients are at a greater risk of developing Afib (24). HF is also associated with increased activity of procoagulant factors and increased thromboembolic events, as well as increased risk of stroke even in the absence of Afib (25–27). Among HF patients without Afib, the stroke risk in patients with or without reduced EF is the same; however, in the presence of Afib, the stroke risk is higher in those with reduced EF than in those with preserved EF (20, 27). In other words, HF in the presence of Afib is associated with 5 times higher odds of stroke compared with 3.5 in patients with HF and no evidence of Afib (28).

Studies on the risk of stroke in HF patients have used different criteria to include HF patients (25). Those investigations utilizing different EF categories have shown that patients with severely reduced EF and high CHA_2_DS_2_-VASc score have a particularly higher risk of ischemic stroke (29, 30). These previous reports support our findings in that severely reduced left ventricular function is associated with higher risk of stroke. Furthermore, we demonstrated significantly higher rates of combined enlarged LA and severely reduced EF among patients with Afib and cardioembolic stroke. Therefore, we propose that the presence of echocardiographic evidence of combined enlarged LA and severely reduced EF (<30%) obviates the necessity for diagnosis of Afib in consideration of anticoagulation for secondary stroke prevention.

Multiple studies have evaluated the benefit of anticoagulation over antiplatelet for stroke prevention in patients with HF and sinus rhythm. Although findings were suggestive of a small benefit with anticoagulation, warfarin was not superior to aspirin due to higher risk of bleeding (31–33).

The NAVIGATE ESUS [New Approach Rivaroxaban Inhibition of Factor Xa in a Global Trial vs. ASA to Prevent Embolism in Embolic Stroke of Undetermined Source (ESUS)] trial showed that in patients with an ESUS, there was no significant difference in stroke recurrence between the rivaroxaban and aspirin groups (34). However, in the predefined subgroup of patients with a LA diameter of more than 4.6 cm, there was a significant reduction in recurrent stroke among patients who were treated with rivaroxaban (1.7 vs. 6.5% per year; hazard ratio, 0.26; 95% CI, 0.07–0.94; *p* = 0.02) (35).

Long-term monitoring with loop recorder for Afib can be very costly. Our results suggested that the combination of LA enlargement with decreased EF is an independent predictor of Afib and cardioembolic stroke and may present an indication for anticoagulation for secondary stroke prevention.

Our study has limitations. First, we excluded a significant number of patients due to presumed lacunar infarcts and the unavailability of echocardiogram, which is most of the time requested as part of the work-up when MRI findings are highly suggestive of embolic type of stroke. We did not include a number of variables, such as body mass index and the presence or absence of valvular disease, which may affect the LA size and left ventricular function. Lastly, this is a single center retrospective study. Further studies are warranted to investigate the association between the combination of enlarged LA with reduced EF with cardioembolic stroke and the benefit of anticoagulation for secondary stroke prevention.

## Data Availability Statement

The raw data supporting the conclusions of this article will be made available by the authors, without undue reservation.

## Ethics Statement

The studies involving human participants were reviewed and approved by University of California Irvine Institutional Review Board. Written informed consent for participation was not required for this study in accordance with the national legislation and the institutional requirements.

## Author Contributions

MH contributed to the concept development, literature review, data and statistical analyses, manuscript draft preparation, and final revision. SM-N contributed to the literature review, data and statistical analyses, and final revision. SL contributed to the data and statistical analyses and final revision. WY contributed to the discussions of important intellectual contents, manuscript draft preparation, critical revision, and final revision. MS contributed to the concept development, data and statistical analyses, manuscript draft preparation, critical revision, and final revision. All authors contributed to the article and approved the submitted version.

## Conflict of Interest

The authors declare that the research was conducted in the absence of any commercial or financial relationships that could be construed as a potential conflict of interest.

## Publisher's Note

All claims expressed in this article are solely those of the authors and do not necessarily represent those of their affiliated organizations, or those of the publisher, the editors and the reviewers. Any product that may be evaluated in this article, or claim that may be made by its manufacturer, is not guaranteed or endorsed by the publisher.

## References

[1] Sacco RL Ellenberg JH Mohr JP Tatemich TK Hier DB Price TR. Infarcts of undetermined cause: the NINCDS Stroke Data Bank. Ann Neurol. (1989) 25:382–90. 10.1002/ana.410250410 2712533

[2] Li L Yiin GS Geraghty OC Schulz UG Kuker W Mehta Z. Incidence, outcome, risk factors, and longterm prognosis of cryptogenic transient ischaemic attack and ischaemic stroke: a population-based study. Lancet Neurol. (2015) 14:903–13. 10.1016/S1474-4422(15)00132-5 26227434 PMC5714616

[3] Ji R Schwamm LH Pervez MA Singhal AB. Ischemic stroke and transient ischemic attack in young adults: risk factors, diagnostic yield, neuroimaging, and thrombolysis. JAMA Neurol. (2013) 70:51–7. 10.1001/jamaneurol.2013.575 23108720

[4] Wolf ME Grittner U Böttcher T sifap1 TM investigators. Phenotypic ASCO characterisation of young patients with ischemic stroke in the prospective multicentre observational sifap1 study. Cerebrovasc Dis. (2015) 40:129–35. 10.1159/000434760 26227782

[5] Galli A Ambrosini F Lombardi F. Holter monitoring and loop recorders: from research to clinical practice. Arrhythm Electrophysiol Rev. (2016) 5:136–43. 10.15420/AER.2016.17.2 27617093 PMC5013174

[6] Sanna T Diener HC Passman RS Lazzaro VD Bernstein RA Morillo CA. Cryptogenic stroke and underlying atrial fibrillation. New Engl J Med. (2014) 370:2478–86. 10.1056/NEJMoa1313600 24963567

[7] Barnes ME Miyasaka Y Seward JB Seward JB Gersh BJ Rosales G. Left atrial volume in the prediction of first ischemic stroke in an elderly cohort without atrial fibrillation. Mayo Clin Proc. (2004) 79:1008–14. 10.4065/79.8.1008 15301328

[8] Xue J Lin Y Chen X Li Q Cai Z Zhang W. Left atrial size and risk of recurrent ischemic stroke in a Chinese population. Brain Behav. (2017) 7:e00702. 10.1002/brb3.702 28523236 PMC5434199

[9] Kaniz F Bailey KR; Petty GW Meissner I Osranek M Alsaileek A. Increased left atrial volume index: potent biomarker for first-ever ischemic stroke. Mayo Clin Proc. (2008) 83:1107–15. 10.4065/83.10.1107 18828970

[10] Kamel H Okin PM Longstreth WT Jr Elkind MSV Soliman EZ. Atrial cardiopathy: a broadened concept of left atrial thromboembolism beyond atrial fibrillation. Future Cardiol. (2015) 11:323–31. 10.2217/fca.15.22 26021638 PMC4868349

[11] Yaghi S Moon YP Mora-McLaughlin C Willey JZ Cheung K Tullio MRD. Left atrial enlargement and stroke recurrence: the Northern Manhattan Stroke Study. Stroke. (2015) 46:1488–93. 10.1161/STROKEAHA.115.008711 25908460 PMC4442058

[12] Benjamin EJ D'Agostino RB Belanger AJ Wolf PA Levy D. Left atrial size and the risk of stroke and death. The Framingham Heart Study. Circulation. (1995) 92:835–41. 10.1161/01.CIR.92.4.835 7641364

[13] Shaikh Q Ahmed B Ahmed M Mahar JH Ahmad M Ahmed A. Left atrial volumes and associated stroke subtypes. BMC Neurol. (2013) 13:149. 10.1186/1471-2377-13-149 24139054 PMC4015833

[14] Alberts VP Bos MJ Koudstaal P Hofman A Witteman JCM Sticker BHC. Heart failure and the risk of stroke: the Rotterdam Study. Eur J Epidemiol. (2010) 25:807–812. 10.1007/s10654-010-9520-y 21061046 PMC2991556

[15] Haeusler KG Laufs U Endres M. Chronic heart failure and ischemic stroke. Stroke. (2011) 42:2977–82. 10.1161/STROKEAHA.111.628479 21903953

[16] Caldwell JC Mamas MA. Heart failure, diastolic dysfunction and atrial fibrillation, mechanistic insight of a complex inter-relationship. Heart Fail Rev. (2012) 17:27–33. 10.1007/s10741-010-9204-4 21103928

[17] Saver JL. Clinical practice. Cryptogenic stroke. New Engl J Med. (2016) 374:2065–74. 10.1056/NEJMcp1503946 27223148

[18] Kim W Kim EJ. Heart failure as a risk factor for stroke. J Stroke. (2018) 20:33–45. 10.5853/jos.2017.02810 29402070 PMC5836579

[19] Kannel WB Wolf PA Verter J. Manifestations of coronary disease predisposing to stroke. The Framingham study. JAMA. (1983) 250:2942–46. 10.1001/jama.1983.03340210040022 6227757

[20] Abdul-Rahim AH Perez AC Fulton RL Jhund PS Latini R Tognoni G. Risk of stroke in chronic heart failure patients without atrial fibrillation: analysis of the controlled Rusovastatin in multi national trial of heart failure (CORONA). Circulation. (2015) 131:1486–94. 10.1161/CIRCULATIONAHA.114.013760 25810334

[21] Pullicino PN Qian M Sacco RL Freudenberger R Graham S Teerlink JR. Recurrrent stroke in the warfarin versus aspirin in reduced ejection fraction (WARCEF) trial. Cerebrovasc Dis. (2014) 38:176–81. 10.1159/000365502 25300706 PMC4245504

[22] Jiamsripong P Honda T Reuss CS Hurst RT Chaliki HP Grill DE. Three meethods for evaluation of left atrial volume. Eur J Echocardiogr. (2008) 9:351–5. 10.1016/j.euje.2007.05.004 17658300

[23] Mogensen UM Jhund PS Abraham WT Desai AS Dickstein K Packer M. Type of atrial fibrillation and outcomes in patients with heart failure and reduced ejection fraction. J Am Coll Cardiol. (2017) 70:2490–500. 10.1016/j.jacc.2017.09.027 29145948

[24] Wang TJ Larson MG Levy D Vasan RS Leip EP Wolf PA. Temporal relations of atrial fibrillation and congestive heart failure and their joint influence on mortality: the Framingham Heart Study. Circulation. (2003) 107:2920–5. 10.1161/01.CIR.0000072767.89944.6E 12771006

[25] Adelborg K Szépligeti S Sundbøll J Horváth-Puhó E Henderson VW Ording A. Risk of stroke in patients with heart failure: a population-based 30-year cohort study. Stroke. (2017) 48:1161–8. 10.1161/STROKEAHA.116.016022 28377383

[26] Lip GY Ponikowski P Andreotti F Anker SD Filippatos G Homma S. Thrombo-embolism and antithrombotic therapy for heart failure in sinus rhythm. A joint consensus document from the ESC Heart Failure Association and the ESC Working Group on Thrombosis. Eur J Heart Fail. (2012) 14:681–95. 10.1093/eurjhf/hfs073 22611046

[27] Abdul-Rahim AH Perez AC MacIsaac RL Jhund PS Claggett BL Carson PE. Risk of stroke in chronic heart failure patients with preserved ejection fraction, but without atrial fibrillation: analysis of the CHARM-Preserved and I-Preserve trials. Eur Heart J. (2017) 38:742–50. 10.1093/eurheartj/ehw509 28426886 PMC5460584

[28] Kang SH Kim J Park JJ Oh IY Yoon CH Kim HJ. Risk of stroke in congestive heart failure with and without atrial fibrillation. Int J Cardiol. (2017) 248:182–7. 10.1016/j.ijcard.2017.07.056 28826798

[29] Freudenberger RS Hellkamp AS Halperin JL Poole J Anderson J Johnson G. Risk of thromboembolism in heart failure: an analysis from the Sudden Cardiac Death in Heart Failure Trial (SCD-HeFT). Circulation. (2007) 115:2637–41. 10.1161/CIRCULATIONAHA.106.661397 17485579

[30] Melgaard L Gorst-Rasmussen A Lane DA Rasmussen LH Larsen TB Lip GYH. Assessment of the CHA2DS2-VASc score in predicting ischemic stroke, thromboembolism, and death in patients with heart failure with and without atrial fibrillation. JAMA. (2015) 314:1030–8. 10.1001/jama.2015.10725 26318604

[31] Homma S Thompson J LP Pullicino PM Levin B Freudenberger RS Teerlink JR. Warfarin and aspirin in patients with heart failure and sinus rhythm. New Engl J Med. (2012) 366:1859–69. 10.1056/NEJMoa1202299 22551105 PMC3723382

[32] Cokkinos DV Haralabopoulos GC Kostis JB Toutouzas PK HELAS investigators. Efficacy of antithrombotic therapy in chronic heart failure: the HELAS study. Eur J Heart Fail. (2006) 8:428–32. 10.1016/j.ejheart.2006.02.012 16737850

[33] Massie BM Krol WF Ammon SE Armstrong PW Cleland JG Collins JF. The Warfarin and Antiplatelet Therapy in Heart Failure trial (WATCH): rationale, design, and baseline patient characteristics. J Cardiac Fail. (2004) 10:101–12. 10.1016/j.cardfail.2004.02.006 15101020

[34] Kasner SE Lavados P Sharma M Wang Y Wang Y Dávalos A. Characterization of patients with embolic strokes of undetermined source in the NAVIGATE ESUS randomized trial. J Stroke Cerebrovasc Dis. (2018) 27:1673–82. 10.1016/j.jstrokecerebrovasdis.2018.01.027 29525076 PMC6701183

[35] Healey JS Gladstone DJ Swaminathan B Eckstein J Mundl H Epstein AE. Recurrent stroke with rivaroxaban compared with aspirin according to predictors of atrial fibrillation: secondary analysis of the NAVIGATE ESUS randomized clinical trial. JAMA Neurol. (2019) 76:764–73. 10.1001/jamaneurol.2019.0617 30958508 PMC6583060

